# The effect of personalized perioperative blood pressure management on intraoperative cerebral oxygen saturation, burst suppression ratio and postoperative neurological outcomes in patients having major non-cardiac surgery: an observational substudy of the IMPROVE-pilot randomized controlled trial

**DOI:** 10.1007/s10877-025-01402-y

**Published:** 2025-12-22

**Authors:** Wiam Khader, Marc Hein, Karim Kouz, Alina Bergholz, Bernd Saugel, Julia Wallqvist, Sebastian Goldmann, Katharina Gräfe, Jan Larmann, Linda Grüßer

**Affiliations:** 1 Department of Anesthesiology, University Hospital RWTH Aachen, Aachen, Germany; 2 Department of Anesthesiology, Center of Anesthesiology and Intensive Care Medicine, University Medical Center Hamburg-Eppendorf, Hamburg, Germany; 3 Alexianer Center for Mental Health, Aachen, Germany

**Keywords:** Blood pressure, Intraoperative hypotension, Neuromonitoring, NIRS, Delirium, Delayed neurocognitive recovery

## Abstract

**Abstract:**

It is not clear whether adopting personalized intraoperative blood pressure management could lead to better intraoperative regional cerebral saturation (rSO2), lower burst suppression ratio (BSR) or better neurological outcomes. Therefore, we performed this prespecified exploratory substudy of the IMPROVE-pilot trial to investigate the effects of personalized compared to routine intraoperative blood pressure management on the intraoperative rSO2. We also explored the effect of personalized intraoperative blood pressure management on BSR and the incidence of postoperative delirium (POD) and delayed neurocognitive recovery (dNCR). We included patients aged ≥ 45 years with American Society of Anesthesiologists (ASA) physical status II-IV who were scheduled for elective major surgery. Preoperative automated nighttime blood pressure measurements were performed. Patients were randomized to personalized blood pressure management maintaining intraoperative mean arterial pressure (MAP) at least at the preoperative mean nighttime MAP or to routine blood pressure management with a lower MAP intervention threshold of 65 mmHg. Intraoperative measurements of MAP, rSO2 on both hemispheres, and BSR were performed. POD was assessed daily on the first 3 postoperative days using the 3D-confusion assessment method or the confusion assessment method for the intensive care unit. We screened for dNCR using the telephone-Montreal Cognitive Assessment on postoperative days 3, 7, and 30. We enrolled 55 patients and randomized 50 patients. 49 patients were included in the final analysis. The median areas under the baseline rSO2 and BSR were similar between the two groups. One patient assigned to personalized blood pressure management and none of the patients assigned to routine blood pressure management had POD. There was no meaningful difference in the incidence of dNCR between the groups. In this substudy of the IMPROVE-pilot trial, we observed no evidence of difference in intraoperative area under baseline rSO2 between patients who received personalized compared to routine perioperative blood pressure management.

**Trial registration:**

This substudy was registered at the German Clinical Trials Register (Deutsches Register Klinischer Studien DRKS00025762, on December 3, 2021).

**Supplementary Information:**

The online version contains supplementary material available at 10.1007/s10877-025-01402-y.

## Introduction

In patients undergoing major surgery, postoperative delirium (POD) and delayed neurocognitive recovery (dNCR) are common [1] and associated with increased mortality, morbidity, and healthcare costs [2]. The main risk factors for postoperative neurocognitive disorders, include age, cognitive status, and patients’ comorbidities [3]. While many of these factors are often challenging to modify in perioperative settings, intraoperative hypotension represents a modifiable risk factor for postoperative neurocognitive disorders that anesthesiologists can actively influence during surgery. Intraoperative hypotension, especially below the lower level of cerebral autoregulation levels, may result in cerebral hypoperfusion with a mismatch between cerebral oxygen demand and supply. This mismatch could trigger neuroinflammation as well as neuronal dysfunction and subsequently promote postoperative neurocognitive disorders [4–8]. However, observational research showed conflicting results on the association between intraoperative hypotension and postoperative neurocognitive disorders [9–13]. It remains unknown whether individualizing intraoperative blood pressure management can help reduce postoperative neurocognitive disorders.

Near-infrared spectroscopy (NIRS) as well as processed electroencephalography (pEEG) can be used to assess surrogate variables for brain perfusion and brain metabolic activity. NIRS allows continuous monitoring of regional cerebral oxygen saturation (rSO2) [14]. Drops in NIRS-derived rSO2 during intraoperative hypotension below the lower limit of cerebral autoregulation reflect compromised cerebral perfusion and oxygen delivery which could increase the risk of postoperative cognitive disorders [15–17]. pEEG monitoring provides a real-time assessment of metabolic brain activity by quantifying electrical patterns that reflect neuronal function and anesthetic depth. The burst suppression ratio (BSR) represents the proportion of time in which the EEG is in a suppressed state, indicating marked metabolic suppression of cortical neurons. Such suppression may occur during episodes of cerebral hypoperfusion, particularly when blood pressure falls below autoregulatory thresholds [18–20].

The role of the brain as an index organ for detecting injury in other organs has been debated. Because cerebral autoregulatory mechanisms tend to preserve cerebral blood flow even at the expense of systemic perfusion, a decrease in rSO2 may indicate a significant systemic circulatory compromise that could result in organ injury such as kidney or heart [21–23].

To date it remains unclear whether individualizing intraoperative blood pressure management strategies affects these neuromonitoring parameters and improves neurological outcome. We therefore performed this prespecified exploratory substudy of the IMPROVE-pilot trial [24] to investigate the effects of personalized compared to routine intraoperative blood pressure management on the intraoperative rSO2. We also explored the effect of personalized intraoperative blood pressure management on BSR and the incidence of POD and dNCR.

## Methods

This study was a prespecified exploratory prospective single-center substudy of the bicentric IMPROVE-pilot trial [24] at the RWTH Aachen University Hospital, Aachen, Germany. This substudy was registered at the German Clinical Trials Register (Deutsches Register Klinischer Studien: DRKS00025762) and conducted between October 4, 2021, and December 24, 2022. Ethical approval was obtained from the Independent Ethical Committee of the Medical Faculty RWTH Aachen (EK 196/20), and written informed consent was obtained from all patients for both the main trial and the substudy prior to inclusion. This substudy is reported in accordance with the CONSORT statement [25].

### Subjects

We included patients aged ≥ 45 years with American Society of Anesthesiologists (ASA) physical status II-IV who were scheduled for elective major surgery, including general, abdominal, gynecology, urology, and trauma surgery, under general anesthesia with an expected surgery duration ≥ 120 min [24]. We excluded patients having emergency surgery or transplant surgery; pregnant patients; patients who had kidney, liver, heart, or lung transplants; patients with sepsis; and patients having surgery requiring controlled hypotension. Further exclusion criteria were a mean arterial pressure (MAP) difference between the right and the left arm of more than 20 mmHg at baseline or any contraindication against preoperative automated nighttime blood pressure measurements.

## Protocol

In the main IMPROVE-pilot trial [24], patients underwent automated nighttime blood pressure measurement prior to surgery to determine their mean nighttime MAP. Patients were randomized in a 1:1 ratio to either personalized or routine blood pressure management using computer-generated codes, with concealment ensured through consecutively numbered opaque envelopes opened shortly before induction of anesthesia. The treating anesthesiologists were instructed to maintain the intraoperative MAP at or above the mean nighttime MAP (with a maximum target of 100 mmHg, or ≥ 65 mmHg if lower) in the personalized blood pressure management group, and above 65 mmHg in the routine blood pressure group. Intraoperative MAP was maintained using routine interventions (e.g., administration of intravenous fluids, vasoactive medication, adjusting depth of anesthesia, positioning of the patient) and was left to the choice of the treating anesthesiologist with no predefined protocol. General anesthesia was maintained with either sevoflurane or propofol in combination with sufentanil or remifentanil. When indicated, epidural catheters were placed before induction.

Study personnel performing follow-up visits were blinded to group allocation. The study personnel collecting intraoperative data and the treating anesthesiologists could not be blinded to group allocation due to the nature of the study. However, the treating anesthesiologists were blinded to intraoperative rSO2 and BSR measurements.

## Measurements

Preoperative automated nighttime blood pressure measurements were performed with the Boso TM-2430 PC2 device (Bosch + Sohn, Jungingen, Germany) and analyzed on the day of surgery using the Boso profile-manager software (Version 4.2.0.133, Bosch + Sohn) according to instructions in the main study [24]. During surgery, patients had continuous intraarterial blood pressure monitoring with an arterial catheter or intermittent oscillometric blood pressure monitoring at 3-minute intervals.

We measured intraoperative rSO2 and the BSR using the Masimo Root device (Version 013) with SedLine^®^ Sedation sensors and O3^®^ regional oximetry sensors (Masimo, Irvine, California, USA). Baseline rSO2 values were obtained for 1–3 min prior to preoxygenation.

rSO2, BSR and intraarterial blood pressure data were collected in 1-second intervals using the MediCollector Bedside software (Version 1.1.29, Winchester, Massachusetts, USA) and the medians over a 1-minute period were calculated for further analyses. Measurements were collected throughout the procedure and ended with the termination of anesthesia, defined as extubation or leaving the operation theater in patients who remained intubated.

## Endpoints

Although this study is exploratory in nature, we pre-defined the following endpoints to reflect the main objectives of this substudy. The primary endpoint was the area under baseline rSO2 on both hemispheric sides in the personalized versus the routine blood pressure management group. Further endpoints were the detection of cumulative BSR duration, the incidence of POD within the first 3 postoperative days, and the incidence of dNCR between postoperative days 3 and 30 in both groups. Furthermore, we calculated areas under baseline rSO2 and rSO2 of 60% in patients who developed myocardial injury and acute kidney injury (AKI) and those who did not within the first 3 postoperative days.

POD was assessed daily on the first 3 postoperative days using the 3D-confusion assessment method or the confusion assessment method for the intensive care unit (CAM-ICU) [26, 27]. dNCR was assessed using the telephone-Montreal Cognitive Assessment (t-MoCa) [28] preoperatively and on postoperative days 3, 7, and 30. A decline of ≥ 3 points in the T-MoCA score was defined as clinically relevant [29]. For each patient, the greatest decrease from baseline observed across the three postoperative assessments was used to determine the presence of dNCR.

Definitions of myocardial injury and AKI and thresholds for their detection were reported in the main IMPROVE-pilot trial [24]: AKI was defined as an increase in postoperative creatinine of ≥ 50% from baseline within the first 3 postoperative days [30], and myocardial injury as a postoperative troponin concentration above the 99th percentile upper reference limit (14 ng/L) with either a ≥ 50% increase from baseline if initially below this limit, or a ≥ 20% increase from baseline if already elevated [31]. In patients with missing baseline creatinine or troponin values, AKI or acute myocardial injury were not assessed. Missing postoperative laboratory values were assumed to be non-pathologic.

### Statistical analysis

No separate sample size calculation was performed for this explorative substudy. The sample size was determined by the number of patients enrolled in the main trial at RWTH Aachen University Hospital. All 50 patients who were enrolled in the IMPROVE-pilot trial at RWTH Aachen University Hospital were also included in this substudy. The analysis was conducted in the modified intention-to-treat population, comprising all patients who were randomized to one of the respective groups and had surgery.

Descriptive statistics were used to describe patients’ characteristics, their relevant clinical data, and exploratory outcomes. Continuous data are presented as mean and standard deviation (mean ± SD) or as median (25th – 75th percentile).

The area under the baseline rSO2 was calculated using the formula published by Mayr et al. [32]:

$$\begin{aligned} {\mathrm{area}}\,{\mathrm{under}}\,{\mathrm{baseline}}\, \text {rSO2}=&\left(\text {baseline rSO2} - \text {current rSO2} \right) \\ &\times \text {time} \text {(sec)}.\end{aligned}$$

The variables across groups were compared using Fisher’s exact test, or Mann–Whitney *U* test as indicated. Effect sizes were reported as estimated difference in location with 95% confidence intervals or as odds ratios with 95% confidence intervals as appropriate. We used SPSS (version 26; IBM, New York, United States) for all statistical analyses. Visualization was conducted using R version 4.5.2 (R Foundation for Statistical Computing, Vienna, Austria).

## Results

**Fig. 1:**
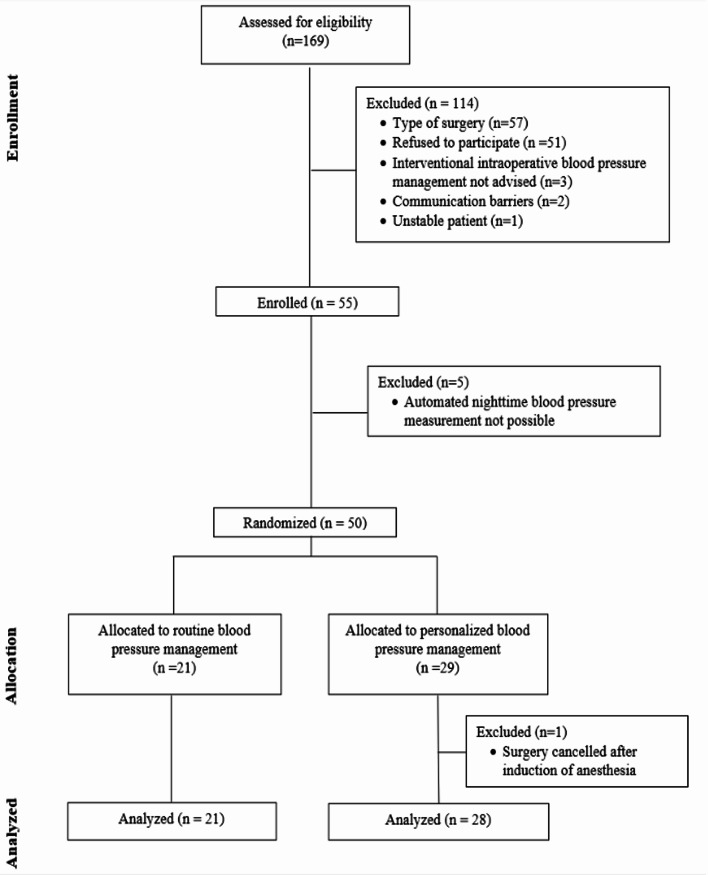
Flow diagram of the study participants including screening, allocation, and reasons of exclusion

The main IMPROVE-pilot trial was terminated after enrolling 105 patients out of 200 planned patients. At our center, we enrolled 55 patients and randomized 50 patients (Fig. 1). For our single-center substudy, we excluded one patient after randomization because surgery was cancelled after anesthetic induction. We thus included 49 patients in the final analysis (Table 1).

**Table 1:** Baseline and perioperative patients’ characteristics

Patient characteristics*	Routine blood pressure management *(n =* 21)	Personalized blood pressure management *(n =* 28)
Age (years)	69.7 ± 11.1	68.3 ± 11.1
Female (n, %)	7 (33)	15 (54)
Height (cm)	173.8 ± 5.4	169.1 ± 10.3
Weight (kg)	84.0 ± 18.6	81.5 ± 19.6
BMI (kg/m^2^)	27.7 ± 5.1	27.8 ± 5.7
Mean nighttime MAP (mmHg)	89 ± 11	87 ± 10
ASA physical status (n, %) II III IV	11 (52) 10 (48) 0	10 (36) 15 (54) 3 (11)
Comorbidities (n, %) Arterial hypertension Diabetes mellitus Heart failure Chronic kidney disease Cardiovascular disease ^a^	12 (57) 7 (33) 1 (5) 0 6 (29)	18 (64) 5 (18) 1 (4) 3 (11) 4 (14)
Medications (n, %) Beta blocker Statins Platelet inhibitors	5 (24) 7 (33) 7 (33)	7 (25) 8 (29) 4 (14)
Operative Risk (n, %) Intermediate risk High risk	3 (14) 18 (86)	10 (36) 18 (64)
Preoperative T-MoCA score	19.1 ± 1.7	19.0 ± 2.9
Duration of anesthesia (min)	271 ± 119	279 ± 115
Intraoperative TWA MAP below 65mmHg (mmHg)	0.45 (0.11–1.22)	0.07 (0.02–0.22)
Intraoperative TWA MAP below mean nighttime MAP (mmHg)	10.47 (5.49–13.49)	3.79 (1.53–7.70)

Categorical data are presented as number (percentage), continuous data as mean ± SD or median (25th −75th percentile) as appropriate. Percentages May not sum up to 100% due to rounding

*No statistical tests were conducted to compare baseline demographic characteristics between the two groups, in accordance with CONSORT guidelines [25]

^a^Cardiovascular disease includes peripheral vascular disease, ischemic heart disease, and stroke

ASA = American Society of Anesthesiologists; MAP = mean arterial pressure; T-MoCA = telephone-Montreal Cognitive Assessment, TWA = time-weighted average

Patients in the personalized blood pressure management group of our substudy, had an intraoperative median time-weighted average (TWA) MAP below mean nighttime MAP of 3.79 (1.53–7.70) mmHg. In the routine blood pressure management group, the TWA MAP below 65 mmHg was 0.45 (0.11–1.22) mmHg.

**Fig. 2:**
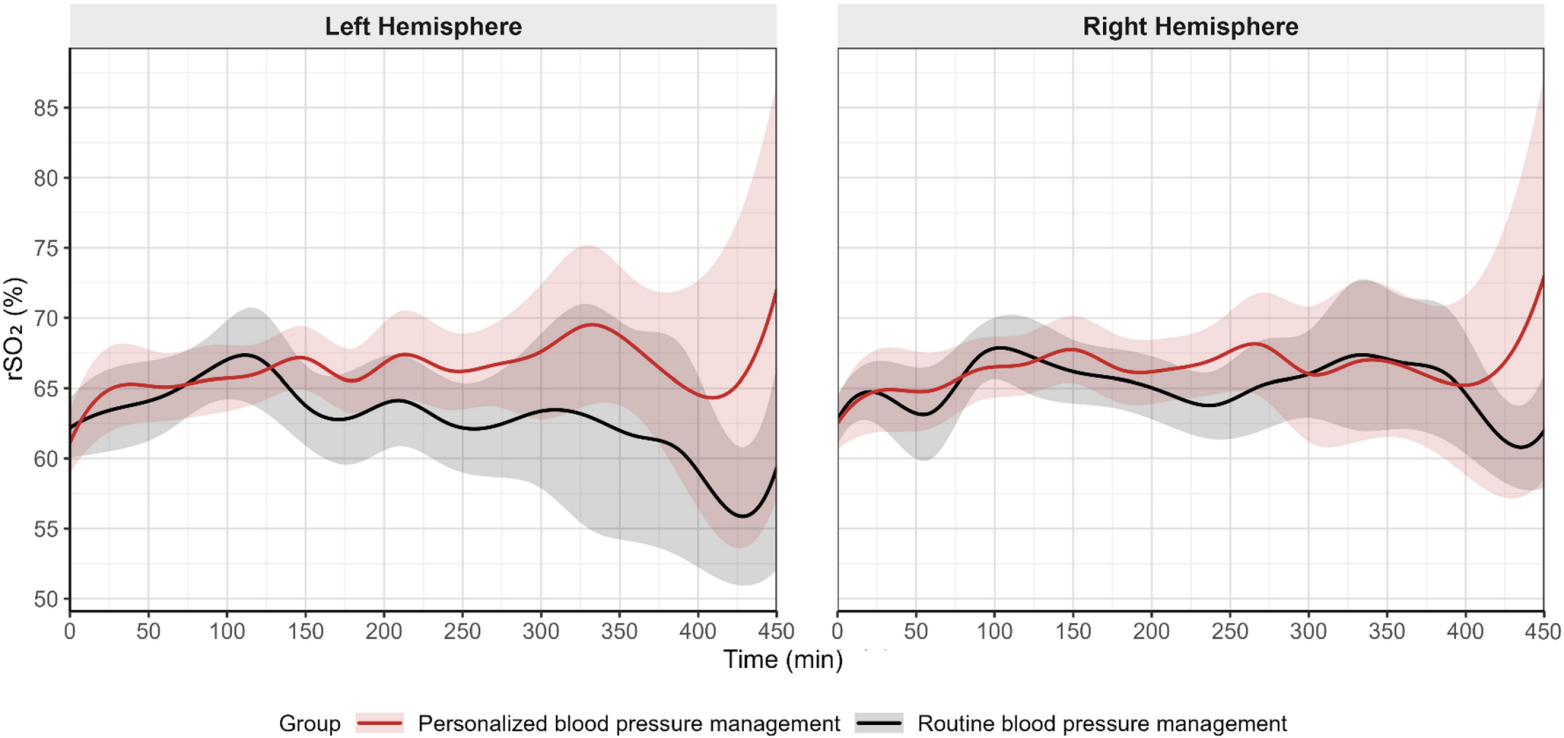
Mean trajectories of rSO2 across the intervention period for the left and right hemispheres. Solid lines represent the mean rSO2 values for the personalized and routine blood pressure management groups; shaded areas denote the corresponding 95% confidence bands

Measurements of rSO2 throughout the intervention period are shown in Fig. 2. The median area under the baseline rSO2 on the left and right hemispheric sides were similar between the two groups; on the left side: 94.5 (2.0–242.0) min% in patients assigned to personalized blood pressure management and 122.0 (18.0–294.0) min% in patients assigned to routine blood pressure management (estimated difference in location (95%-CI): 22.5 (−35.0–129.0) min%, *P* =.367); on the right side: 42.0 (1.0–255.0) min% *versus* 23.0 (4.0–291.0) min% (estimated difference in location (95%-CI): 0.0 (−139.0–51.0) min%, *P* =.983).

There was no relevant difference in the median BSR duration between the groups.

(5.69 (0.57–17.48) min in the personalized blood pressure management group *versus* 1.17 (0.39–8.92) min in the routine blood pressure management group, estimated difference in location (95%-CI): −2.4 (−8.1–0.4) min, *P* =.140).

One patient assigned to personalized blood pressure management (3.5%) and none of the patients assigned to routine blood pressure management had POD. Between postoperative day 3 and 30, six patients in the personalized blood pressure management group (21%) and one patient in the routine blood pressure management group (5%) had dNCR (Table 2).

rSO2 data for patients with POD and dNCR are shown in supplementary materials S1, Tables S1-S2.

**Table 2:** Exploratory outcomes of the IMPROVE-pilot substudy

Characteristics	Routine blood pressure management *(n =* 21)	Personalized blood pressure management *(n =* 28)	Effect size* (95% CI)	*P*-Value
Area under baseline rSO2 left (min%)	122.0 (18.0–294.0)	94.5 (2.0–242.5)	22.5 (−35.0–129.0)	0.367^f^
Area under baseline rSO2 right (min%) ^a^	23.0 (4.0–291.0)	42.0 (1.0–255.0)	0.0 (−139.0–51.0)	0.983^f^
BSR (min)	1.17 (0.39–8.92)	5.69 (0.57–17.48)	−2.4 (−8.1–0.4)	0.140^f^
Postoperative delirium (n, %)^b^	0	1 (3.5)		1.00^g^
dNCR between postoperative day 3 and 30 (n, %)^c, d,e^	1 (5)	6 (21)	5.7 (0.6–51.9)	0.119^g^
T-MoCa score at postoperative day 3^c^	20.0 (18.0–21.0)	21.0 (17.50–22.0)	−1 (−2.0–1.0)	0.358^f^
T-MoCA score at postoperative day 7^d^	20.0 (17.50–21.0)	21.0 (18.25–22.0)	0.0 (−1.0–2.0)	0.899^f^
T-MoCa score at postoperative day 30^e^	19.0 (17.0–22.0)	21.5 (17.25–22.0)	−1 (−3.0–1.0)	0.346^f^

Categorical data are presented as number (percentage), continuous data as median (25th-75th percentile)

*Effect sizes for continuous variables analyzed with the Mann–Whitney U test are reported as the estimated difference in location with 95% confidence intervals. Effect sizes for categorical variables are expressed as odds ratios with 95% confidence intervals and statistical significance was assessed using Fisher‘s exact test

^a^1 patient with missing data on the right hemisphere (in the personalized blood pressure management group)

^b^3 patients with missing data (1 in the routine blood pressure management group and 2 in the personalized blood pressure management group)

^c^11 patients with missing data on day 3 (3 patients in the routine blood pressure management group and 8 patients in the personalized blood pressure management group)

^d^9 patients with missing data on day 7 (5 patients in the routine blood pressure management group and 4 in the personalized BP management group 4)

^e^17 patients with missing data on day 30 (7 patients in the routine blood pressure management group and 10 in the personalized blood pressure management group)

^f^Mann-Whitney U test

^g^Fischer’s exact test

BSR = burst suppression ratio; CI = confidence interval; dNCR = delayed neurocognitive recovery; T-MoCA = telephone-Montreal Cognitive Assessment, rSO2 = regional oxygen saturation index

Seven patients (33%) in the routine blood pressure management group and 14 (50%) patients in the personalized blood pressure management group developed myocardial injury. One patient in each group developed AKI. The median area under baseline rSO2 as well as the median area under a threshold of 60% were slightly higher in patients with myocardial injury compared to patients without myocardial injury (Table 3).

**Table 3:** Comparison of area under baseline rSO2 as well as under rSO2 of 60% between patients who had myocardial injury and those who did not

Characteristics^a^	Patients with myocardial injury (*n* = 21)	Patients without myocardial injury (*n* = 27)^b^
Area under baseline rSO2 left (min%) Area under baseline rSO2 right (min%)^c^	104.0 (10.0–271.0) 88.0 (2.0–263.0)	78.0 (3.0–247.0) 23.0 (1.0–255.0)
Area under rSO2 of 60% left (min%)^d^ Area under rSO2 of 60% right (min%)^c,e^	30.0 (0.0–575.0) 10.5 (0.0–143.5)	4.0 (0.0–92.0) 0.0 (0.0–117.0)

Data presented as median (25th – 75th percentile)

^a^1 patient with missing baseline laboratory values (in the routine blood pressure management group)

^b^1 patient with missing troponin values on postoperative day 2 in the personalized blood pressure management group; 4 patients with missing troponin values on postoperative day 3 (3 patients in personalized blood pressure management group and 1 in the routine blood pressure management group)

^c^1 patient with missing data on the right hemisphere (in the personalized blood pressure management group)

^d^30 patients had drops of rSO2 below 60% on the left hemispheric side (19 in the personalized blood pressure management group and 11 in the routine blood pressure management group). Of those, 16 patients had myocardial injury (10 patients in the personalized blood pressure management group versus 6 patients in the routine blood pressure management group)

^e^26 patients had drops of rSO2 below 60% on the right hemispheric side (15 in the personalized blood pressure management group and 11 in the routine blood pressure management group). Of those, 13 patients had myocardial injury (7 patients in the personalized blood pressure management group and 6 patients in the routine blood pressure management group)

rSO2 = cerebral regional oxygen saturation

## Discussion

In this exploratory substudy of the IMPROVE-pilot trial, we found no clinically relevant differences between the routine and personalized blood pressure management groups regarding areas under baseline rSO2, BSR duration, or the occurrence of POD and dNCR among patients undergoing major noncardiac surgery.

According to the protocol of the main study, the preoperative mean nighttime MAP was used in the personalized blood pressure management group to define the individualized lower intervention thresholds. Compared with isolated daytime or office measurements nocturnal blood pressure is less likely to be influenced by transient external factors such as physical activity, or emotional stress [33], making it a more reliable reflection of a patient’s baseline hemodynamics. Healthy individuals typically exhibit a physiological dipping pattern during sleep, caused by increased vagal tone and reduced sympathetic activity, resulting in lower nocturnal MAP values [34]. Blood pressure, however, varies among individuals [35]. It can be assumed that patients tolerate their individual mean nighttime MAP also during surgery, making it a reasonable reference point for this study.

In the personalized blood pressure management group, the median TWA below mean nighttime MAP was approximately 4 mmHg. To put this into perspective, as described in the main trial [24], a patient with a mean nighttime MAP of 100 mmHg who maintained an intraoperative MAP of 96 mmHg throughout anesthesia would have a TWA of 4 mmHg. This suggests that, despite having higher intraoperative MAP targets, patients in the personalized blood pressure management group were sufficiently kept at their personalized target MAP. Aligning with our findings, other small studies in non-cardiac surgery patients showed that a decrease of up to 20% from baseline blood pressure values or a controlled hypotension with MAP target of 60 to 65 mmHg did not result in a significant reduction in rSO2 levels [36, 37]. On the other side, targeting higher intraoperative blood pressure values did not lead to an increase in rSO2 values either [38]. A possible explanation for the absence of a detectable effect of blood pressure on cerebral saturation could be a functioning autoregulation mechanism that limits the effect of hypotension on brain perfusion [39, 40]. Moreover, both inhalational anesthetics as well as propofol reduce the cerebral metabolic rate of oxygen [41–43], which may offer protection against reduced supply.

Interestingly, we noted that the drops in rSO2 values on the left and right hemispheric sides appeared asymmetric, with larger drops on the left hemispheric side. Although a significant difference in interhemispheric rSO2 was also observed in healthy individuals [44], others argued that large interhemispheric asymmetries in the perioperative setting could be a factor to predict postoperative memory decline or visual-motor coordination and executive function [45]. The positioning of the patient during the operation could also lead to dissymmetry, with higher values in the upper hemisphere when the patient is in a lateral position [46]. However, all patients in our study were operated in the supine position. More research is recommended to understand the significance and clinical relevance of perioperative interhemispheric rSO2 differences.

BSR reflects cortical metabolic suppression due to reduced cerebral metabolic rate, most commonly seen during deep general anesthesia but also occurring in conditions such as hypothermia, hypoxia, or coma [47]. Low blood pressure could result in reduced brain perfusion, leading to ischemic suppression of brain metabolism. This means that a low MAP could be a potential cause of burst suppression [48]. It was observed that lower intraoperative MAP below the brain’s lower limit autoregulation leads to lower bispectral index (BIS) values [49]. While BIS and BSR are not identical parameters (BIS is a proprietary composite index while BSR directly quantifies cortical suppression) it is likely that very low BIS values correspond with higher BSR and thus more cortical metabolic suppression. Intraoperative strategies aiming to increase the MAP to preoperative baseline values could result in a reduction of the BSR duration [50]. In our study we found that targeting higher MAP in the personalized blood pressure management group did not result in clinically relevant BSR duration differences between the two groups. The median intraoperative BSR was even higher in the personalized blood pressure management group compared with the routine blood pressure management group. Importantly, it should be noted that BSR is influenced by multiple perioperative factors beyond blood pressure, particularly type and concentration of the anesthetic agent as well as patients’ characteristics such as age [51, 52].

The relationship between intraoperative hypotension and development of postoperative neurological dysfunction is complex, with studies showing conflicting results [10, 11, 13, 53]. A systematic review and meta-analysis found no association between intraoperative hypotension and postoperative cognitive dysfunction but showed an association with POD and stroke [54]. A recent large randomized clinical trial comparing a perioperative hypotension-avoidance (target MAP of 80 mmHg and higher) versus a hypertension-avoidance strategy (target MAP of 60 mmHg and higher) identified no differences in neurocognitive outcomes between the two strategies [55]. Notably, blood pressure targets were not personalized in this trial. In a smaller trial, personalized intraoperative blood pressure management did not reduce the incidence of POD or dNCR between the third and seventh postoperative days either [56]. This is consistent with our findings, which showed no evidence of difference between the incidences of POD within the first 3 postoperative days or dNCR between postoperative days 3 and 30 in the routine or personalized blood pressure management groups.

The absence of improved rSO2, BSR and neurological outcome in the personalized blood pressure management group may reflect the effect of confounding factors, such as variations in anesthetic depth, or the use of vasoactive medications, which could have counteracted the potential effect of higher MAP targets on cerebral perfusion. This underscores the complexity of interpreting rSO2 and BSR responses in the perioperative setting and highlights the need for larger studies that can account for these interacting variables.

Beyond the open questions regarding the relationship between blood pressure, rSO2, BSR, and neurological outcomes, it remains to be determined whether NIRS and pEEG monitoring, and the identification of specific thresholds can help prevent POD or dNCR in noncardiac surgery patients. While we observed no evidence of differences in BSR duration between the group that developed POD and dNCR and those who did not (Supplementary materials S1, Tables S1-S2), the clinical significance of intraoperative BSR remains a topic of debate: several studies report an association to POD and early postoperative cognitive decline [54, 55], whereas others have proposed that reduced cerebral metabolism during burst suppression may provide a protective effect against neuronal injury and postoperative cognitive disorder [56].

While studies in patients undergoing cardiac surgery found an association between POD and postoperative cognitive dysfunction and cerebral desaturation [57], findings in noncardiac surgery populations remain inconclusive due to substantial heterogeneity across studies [58]. Our small sample size and the limited number of patients who developed POD or dNCR, make it difficult to determine whether the drops in rSO2 correlate with the development of these neurological outcomes in our substudy.

We noticed that patients who had myocardial injury appeared to have a larger area under baseline rSO2 as well as below threshold of 60%. The brain is a highly hypoxia-sensitive organ with autoregulatory mechanisms to maintain a consistent oxygen supply, even under low-supply conditions; it is therefore reasonable to assume that a detected decline in cerebral oxygen supply likely indicates that supply to other organs, including the heart, already occurred. A meta-analysis argued that this autoregulatory mechanism is the reason the brain does not qualify as a proxy organ for injury detection in other organs. However, the effect of the severity or duration of these drops were not investigated [59]. Assessing the area under baseline rSO2 and under specific thresholds in larger future studies may help better understand how the duration and depth of desaturation episodes relate to other organ injury beyond the brain. Regarding organ injury, in the adequately powered multicenter IMPROVE-multi trial, individualized perioperative blood pressure management with MAP targets based on preoperative mean nighttime MAP did not decrease the composite outcome of acute kidney injury, acute myocardial injury, nonfatal cardiac arrest, or death within the first 7 postoperative days compared with routine blood pressure management with a MAP target of 65mmHg or higher [60]. The IMPROVE-multi trial did not focus on the effects of individualized intraoperative blood pressure on neuromonitoring or neurocognitive outcomes.

Overall, larger-scale studies with standardized anesthetic management protocols are outstanding to investigate the associations among intraoperative hemodynamics, intraoperative rSO2, BSR, and postoperative neurological outcomes. The low rates of missing neuromonitoring data in this study demonstrate that this is feasible. Higher rates of missing data for POD and dNCR suggest that the available tools to detect these conditions do not encourage patients’ participation in the postoperative setting.

### Limitations

The most important limitation is the small sample size. Recruitment was slowed by cancellations of elective surgeries during the COVID-19 pandemic, and the main trial was terminated before the planned sample size was reached. However, even without this disruption, this exploratory substudy would have remained underpowered to detect clinically meaningful differences in outcomes. For example, with only a single case of delirium observed, the risk of type II error is considerable, and null findings should not be interpreted as evidence of no effect. In addition, as a single-center substudy, the findings may be influenced by local practice patterns and are therefore paired with limited generalizability. Moreover, screening for POD was performed only during the first 3 postoperative days, which may have missed late-onset cases. We used the T-MoCA to screen for dNCR. This represents a compromise between feasibility and depth of cognitive assessment. While more comprehensive neuropsychological testing may allow a more differentiated evaluation, its use in the perioperative setting is limited by patient burden and compliance. The missing data, especially regarding POD and dNCR, introduces the potential for bias. Finally, reliance on machine-derived parameters (Masimo-derived BSR) may have underestimated BSR duration, as experienced neurologists’ visual assessment of raw EEG may better detect suppression patterns than automated algorithms [61]. Therefore, our results should be interpreted with caution.

## Conclusion

In this substudy of the IMPROVE-pilot trial, no evidence of difference was observed in intraoperative areas under baseline rSO2, BSR, and the incidence of POD within the first 3 postoperative days and dNCR between postoperative days 3 and 30 in patients who received personalized compared to routine perioperative blood pressure management. Large multicenter trials with standardized protocols, extended delirium monitoring, and sensitive neurocognitive testing are needed to clarify the role of personalized blood pressure management on rSO2, BSR and postoperative neurological outcomes.

## Supplementary Information

Below is the link to the electronic supplementary material.

Supplementary Material 1

## Data Availability

No datasets were generated or analysed during the current study.
