# Supplementary material for: The effect of personalized perioperative blood pressure management on intraoperative cerebral oxygen saturation, burst suppression ratio and postoperative neurological outcomes in patients having major non-cardiac surgery: an observational substudy of the IMPROVE-pilot randomized controlled trial
Source: J Clin Monit Comput. 2025 Dec 22;40(2):363–73. doi: 10.1007/s10877-025-01402-y (PMC13053527; doi:10.1007/s10877-025-01402-y)
Supplement: Supplementary file 1 — Supplementary Material 1 [file 10877_2025_1402_MOESM1_ESM.docx]

**Supplementary material S1.**

This supplementary document includes tables with details about area under rSO2 curves and BSR stratified by presence or absence of postoperative delirium (POD) within the first 3 postoperative days, as well as presence and absence of delayed neurocognitive recovery (dNCR) between postoperative days 3 and 30.

| Characteristics | | Routine blood  pressure management  *(n=*21) | Personalized blood  pressure management  *(n =*28) | | | |
| --- | --- | --- | --- | --- | --- | --- |
|  | POD  (n=0)^a^ | No POD  (n=20)^a^ | POD  (n=1)^a^ | | | No POD  (n=25)^a^ |
| Area under baseline left (min%) | - | 105.0 (13.50 - 282.50) | | 134.0 (-) | 104.0 (1.0 -247.0) | |
| Area under baseline right (min%) | - | 35.50 (2.50 - 391.1) | | 218.0 (-) | 107.0 (1.0 - 311.0) | |
| BSR (min) | - | 1.26 (0.43 – 8.92) | | 9.64 (-) | 5.04 (0.57 – 12.41) | |
| Area under rSO2 of 60% left (min%) | - | 3.50 (0.0 - 347.50) | | 384.0 (-) | 26.0 (0.0 - 83.0) | |
| Area under rSO2 of 60% right (min%) | - | 2.50 (0.0 - 145.0) | | 531.0 (-) | 3.50 (0.0 - 119.50) | |

**Table S1**. Comparison of area under baseline rSO2 and under threshold of 60% between routine and personalized blood pressure managements groups in patients who developed POD and those who did not. Data presented as median (25h – 75^th^ percentile).

^a^ 3 Patients with missing data (1 in the routine blood pressure management group and 2 in the personalized blood pressure management group).

BSR = burst suppression Ratio; POD= postoperative delirium; rSO2 = regional oxygen saturation index.

| Characteristics | Routine blood  pressure management  *(n=*21) | | Personalized blood  pressure management  *(n =*28) | | |
| --- | --- | --- | --- | --- | --- |
|  | dNCR  (n=1)^a^ | No dNCR  (n=19)^a^ | dNCR  (n=6)^a^ | No dNCR  (n=20)^a^ | |
| Area under baseline left (min%) | 0.0 (0.0 -0.0) | 122.0 (18.0 – 357.0) | 102.0 (62.0 – 143.0) | 91.0 (0.0 – 268.5) |  |
| Area under baseline right (min%) ^b^ | 6.0 (6.0 – 6.0) | 23.0 (1.0 – 491.0) | 235.0 (5.0 – 367.0) | 37.5 (1.0 – 240.0) |  |
| BSR (min) | 0.4 (0.4 – 0.4) | 1.2 (0.4 – 8.8) | 9.3 (5.0 – 21.1) | 3.2 (0.4 – 13.9) |  |
| Area under rSO2 of 60% left (min%) ^c^ | 0.0 (0.0 -0.0) | 49.0 (0.0 – 575.0) | 32.5 (0.0 – 210.0) | 25.0 (0.0 – 76.0) |  |
| Area under rSO2 of 60% right (min%) ^b,d^ | 0.0 (0.0 -0.0) | 20.0 (0.0 – 170.0) | 79.0 (0.0 – 118.0) | 3.5 (0.0 – 100.5) |  |

**Table S2**. Comparison of area under baseline rSO2 and under threshold of 60% between routine and personalized blood pressure managements groups in patients who developed dNCR between postoperative days 3 and 30 and those who did not. Data are presented as median (25h – 75^th^ percentile).

^a^ Concerning the assessment of the postoperative day 3: 11 patients with missing data (3 patients in the routine blood pressure management group and 8 patients in the personalized blood pressure management group). Concerning the assessment of the postoperative day 7: 9 patients with missing data (5 patients in the routine blood pressure management group and 4 in the personalized blood pressure management group 4). Concerning the assessment of the postoperative day 30: 17 patients with missing data (7 patients in the routine blood pressure management group and 10 in the personalized blood pressure management group).

^b^ 1 patient with missing data on the right hemisphere (in the personalized blood pressure management group).

^c^ 30 patients had drops of rSO2 below 60% on the left hemispheric side (19 in the personalized blood pressure group and 11 in the routine blood pressure group).

^d^ 26-six patients had drops of rSO2 below 60% on the right hemispheric side (15 in the personalized blood pressure group and 11 in the routine blood pressure group).

BSR = burst suppression ratio; dNCR= delayed neurocognitive recovery; rSO2 = regional oxygen saturation index.
